# Current use of intraosseous infusion in Danish emergency departments: a cross-sectional study

**DOI:** 10.1186/1757-7241-18-37

**Published:** 2010-07-01

**Authors:** Rune Molin, Peter Hallas, Mikkel Brabrand, Thomas Andersen Schmidt

**Affiliations:** 1 Department of Emergency Medicine, Holbæk Sygehus, Holbæk, Denmark; 2 Department of Anaesthesiology, JMC, Rigshospitalet, Copenhagen, Denmark; 3 Department of Medicine, Sydvestjysk Sygehus, Esbjerg, Denmark

## Abstract

**Background:**

Intraosseous infusion (IOI) is recommended when intravenous access cannot be readily established in both pediatric and adult resuscitation. We evaluated the current use of IOI in Danish emergency departments (EDs).

**Methods:**

An online questionnaire was e-mailed to the Heads of Department of the twenty EDs currently established in Denmark. The questionnaire focused on the use of IOI in the EDs and included questions on frequency of use, training, equipment and attitudes towards IOI.

**Results:**

We received a total of 19 responses (response rate of 95%). Of the responding 19 Danish EDs 74% (n = 14) reported having intraosseous devices available. The median number of IOI procedures performed in these departments over the preceding 12 months was 5.0 (range: 0-45). In 47% (n = 9) of the departments, prior training sessions in the use of intraosseous devices had not been provided, and 42% (n = 8) did not have local guidelines on IOI. The indication for IOI use was often not clearly defined and only 11% (n = 2) consistently used IOI on relevant indication. This is surprising as 95% (n = 18) of responders were aware that IOI can be utilized in both pediatric and adult resuscitation.

**Conclusions:**

The study shows considerable variations in IOI usage in Danish EDs despite the fact that IOI devices were available in the majority of EDs. In addition, in many EDs there were no local guidelines on IOI and no training in the procedure. We recommend more extensive training of medical staff in IOI techniques in Danish EDs.

## Background

Intraosseous infusion (IOI) has been used during the course of numerous years as a method of delivering drugs and fluids to the vascular system via the bone marrow. The earliest reports of IOI usage was in 1922 by Drinker et al [1] who found that fluids infused into the bone marrow was quickly absorbed into the central circulation. During World War II IOI was used by military doctors for resuscitation [2] but after the war the use of IOI declined considerably [3].

Since the late 1980s the American Heart Association has recommended the use of IOI in pediatric resuscitation [4,5]. IOI is now also recommended in adult resuscitation by Advanced Trauma Life Support (ATLS) [6], the European Resuscitation Council [7] and the American Heart Association [8].

Awareness of the merits of IOI use could potentially direct efforts at increasing adherence to guidelines and quality of care. However, it is not documented to what extent IOI is used in Danish emergency departments (EDs). Our null hypothesis was that IOI is rarely used in this setting. We therefore conducted a cross-sectional study in order to document current use of IOI in Danish EDs.

## Methods

This survey was conducted in January 2010; the information obtained covers the previous 12 months period. We directed the study towards the Danish EDs that accept walk-in patients.

EDs were included in the survey if they:

I. Provided first-line treatment for both medical and surgical patients AND

II. Accepted walk-in patients AND

III. Were located in Denmark (Greenland and Faeroe Islands not included) AND

IV. Had physicians on call

EDs were excluded if

I. They primarily accepted secondary referrals and/or secondary transfers

In effect, the criteria excluded some trauma centers that mainly received patients who had already been attended to by a doctor from the pre-hospital emergency medicine services or doctors from other hospitals. Thus, the study focuses on IOI use in "typical" Danish Emergency Departments, not highly specialized centers. Patients admitted at highly specialized trauma centers by other doctors would be expected to have an IOI established before referral if needed.

Departments were identified using the Danish Healthcare Organization Register and Hospital Department Classification (The Danish Healthcare Organization, personal communication). Twenty departments met the criteria and were included in this study (18 regional hospitals and 2 university hospitals).

The questionnaire (additional file 1) was pilot-tested by using four responders from departments in two hospitals. The status of IOI use in these departments was known to the authors, and we were therefore able to verify the answers in the pilot-test: We found that the responders answered in accordance with the intent of the questionnaire.

The questionnaires were e-mailed to the Heads of Department. Questionnaires not returned within a week, were followed up by telephone calls requesting the responder to complete the survey by telephone. The questionnaire contained items on the total use of IOI in the ED. The IOIs listed can therefore have been placed by physicians from other departments who may have attended patients in the ED.

Due to the design of the study, approval by the ethics committee was not required. The study had undergone institutional review for approval.

Data are presented descriptively. Numerical variables were summarised using median and range. Categorical data were presented as frequencies (percentage).

## Results

We received a total of 19 responses (response rate of 95%). All responders were senior consultants or consultants responsible for training of the ED medical staff. The responding departments in this survey attended an average of 32,000 patients annually (range: 12,000-58,800).

IOI devices were available in 74% (n = 14) of the EDs.

**Figure 1:**
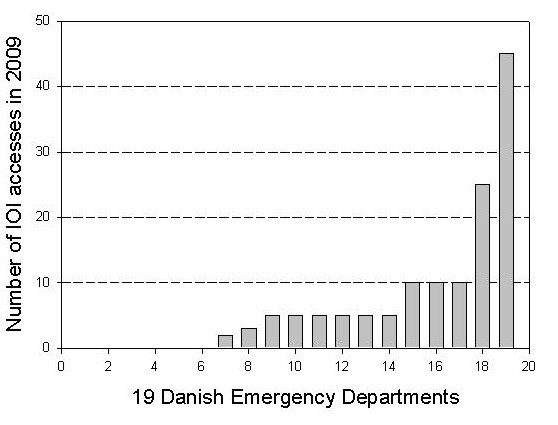
**The number of established IOI accesses in each Emergency Department within the last 12 months**.

There was a large variation in the number of IOIs performed over the preceding 12 months (figure 1). The median number of IOI procedures performed was 5.0 (range: 0-45). The two university hospitals had used IOI 10 and 25 times respectively. In the majority of departments 58% (n = 11), there were no local guidelines for IOI, and in 47% (n = 9) no prior training sessions on IOI use had been provided.

The majority of responders (95% (n = 18)) were aware that IOI could be employed in the resuscitation of the adult patient. However, as seen in table 1, there is no general consensus on the indications for IOI. It is noted that 37% (n = 7) of responders declined the use of IOI before attempts performed by an anesthesiologist, failing to establish intravenous access.

**Table 1:** Responders indications for IOI use

Tick box options*	N	(%)
We never use IOI before anaesthesiologists have tried and failed intravenous access	7	(37%)
Critically ill patients	11	(58%)
Cardiac arrest	7	(37%)
Sepsis shock	8	(42%)
Pulmonary embolism	6	(32%)
Hypovolemic shock	11	(58%)
Other conditions not mentioned above**	5	(26%)

* All options apply when intravenous attempts have failed. It was possible for responders to mark more than one tick box.

**Other conditions mentioned by the responders are: as primary care access to patients with obesity in cardiac arrest, drug addicts, pre-hospital patients, trauma patients and generally in cases with the critically ill patient where an intravenous access cannot be establish within 60 seconds.

There was a lack of consensus as to the contraindications to IOI: 58% (n = 11) reported infusion through a fractured bone as a contraindication, 53% (n = 10) insertion through infected skin, 47% (n = 9) lack of training in IOI, 21% (n = 4) lack of practical experience with IOI and 21% (n = 4) vascular access via alternative methods not previously attempted (e.g. central venous catheter).

The EZ-IO^® ^was the favored IOI device in Danish EDs. Among the departments that had IOI devices 95% (n = 18) had selected EZ-IO^® ^as standard IOI device, 11% (n = 2) had both EZ-IO^® ^and Cook Surfast^® ^and 5% (n = 1) had the Bone Injection Gun^® ^(B.I.G).

The preferred injection sites were tibia (84%, n = 16), humerus (10%, n = 2), medial malleolus (10%, n = 2) and 5% (n = 1) had no preference.

Information on the perceived allocation of tasks and responsibilities was obtained by asking responders whom they expected to operate IOI devices. In descending order it was expected by 78% (n = 15) that IOI handling was performed by the attending anesthesiologist, by 26% (n = 5) the senior resident at the emergency department, by 16% (n = 3) an orthopedic surgeon and by 10% (n = 2) a cardiologist was expected to operate the IOI devices. In 21% (n = 4) of the EDs, staff members expected to perform access using IOI devices, were not clearly identified. None of the responders expected physicians below specialist level to handle IOI access.

One third of the responders (n = 6) were aware of one or more incidents where IOI was indicated but not established. All, but one, were adult patients where several attempts of establishing intravenous access had failed and IOI was not possible. Possible initiatives to promote IOI use in resuscitation were presented to responders as tick box options. Options and answers are shown in table 2.

**Table 2:** Responders opinions on how to promote IOI use

Tick box options*	Yes		No	
	N	(%)	N	(%)
A change in procedure is not needed	4	(21%)	15	(79%)
The indication is so rare, that I see no further need to promote IOI use	4	(21%)	15	(79%)
Training at medical school	3	(16%)	16	(84%)
Training during internship	6	(32%)	13	(68%)
Training during the first year of specialist training	12	(63%)	7	(36%)
Training after first year specialist training	12	(63%)	7	(36%)
The manufacturers should promote their devices	3	(16%)	16	(84%)
Each doctor should alone seek knowledge to perform the procedures	1	(5%)	18	(95%)
Training in IOI should be held by the Emergency Department	12	(63%)	7	(36%)

* It was possible for the responders to give more than one reply.

## Discussion

The study shows considerable variations in IOI usage in Danish EDs despite the fact that IOI devices were available in the majority of EDs. In addition, in many EDs there were no local guidelines on IOI and no training in the procedure.

There are several potential limitations to this survey. Foremost, this is a retrospective study and not all departments keep databases with registration of IOI use. In this situation the responders had to estimate the number of IOI infusions and this could infer recall bias. In addition, the questionnaire was pilot tested on a relatively small number of people. Finally, some trauma centers were not included. Reported use of IOI would probably be higher had they been included in the study. However, the scope of the study was to determine the use of IOI in the typical Danish ED, not in highly specialized centers.

The variations in IOI usage can not be explained convincingly by case mix. The EDs surveyed in the current study provide services to a uniform population and all attended to patients requiring medical or surgical attention. In addition, a previous study of IOI use in adults in the United Kingdom showed a similar pattern as in this study: IOI was both infrequently taught and used in the EDs [9]. The authors recommended a more widespread teaching of the IOI procedure as a way of increasing IOI use in adults. Training in IOI techniques is part of the European Curriculum for Emergency Medicine [10] and training in the subspecialty of emergency medicine in Denmark [11]. Lack of training in IOI use in Danish EDs, indicates an opportunity to improve the training of junior doctors in the EDs.

Skill level present at the studied institutions might influence the number of IOIs. Junior doctors are often front-line personnel in Danish EDs even when seriously ill patients are admitted [12] The current study, however, shows that the Heads of Department are unlikely to expect the establishment of IOI access performed by lower-ranking doctors, despite the procedure being indicated. This could imply that repeated attempts at intravenous access are conducted, rather than using IOI.

Previous practical IOI-experience diminishes the reluctance of paediatricians to the use of IOI in emergencies [13]. Perhaps this could explain why some EDs use IOI up to a factor of nine times more frequently than the mean: Some EDs may simply use IOI frequently due to medical staff becoming accustomed to the application of the procedure of IOI through repetition in incident patient-cases. If this mechanism is in effect, it is unfortunate that more than a third of the responders maintain that IOI-training should not take place in EDs (table 2).

Efforts aimed at increasing IOI-use in accordance with established guidelines, should further address the study of factors related to local variations in the application of IOI procedures.

## Conclusions

IOI is a technique which is reported to be both infrequently taught and used in Danish emergency departments. In many emergency departments IOI was not used at all, and departments that did use IOI often, did not follow indications for IOI use in international recommendations.

The survey suggests a need for training in the use of IOI at many Danish emergency departments

## Competing interests

The authors declare that they have no competing interests.

## Authors' contributions

RM designed the study and prepared manuscript, figure and tables. PH designed the on-line questionnaire. RM, PH, MB conducted data collection. PH, MB, TAS contributed to the study design and added significant revisions. TAS participated as expert instructor and contributed to the study design. All authors participated in drafting, revising and finally approved the article.

## Supplementary Material

Additional file 1 **Questions from the questionnaire**. Click here for file
