# Supplementary figures and images for: Current use of intraosseous infusion in Danish emergency departments: a cross-sectional study
Source: Scand J Trauma Resusc Emerg Med. 2010 Jul 1;18:37. doi: 10.1186/1757-7241-18-37 (PMC2904266; doi:10.1186/1757-7241-18-37)

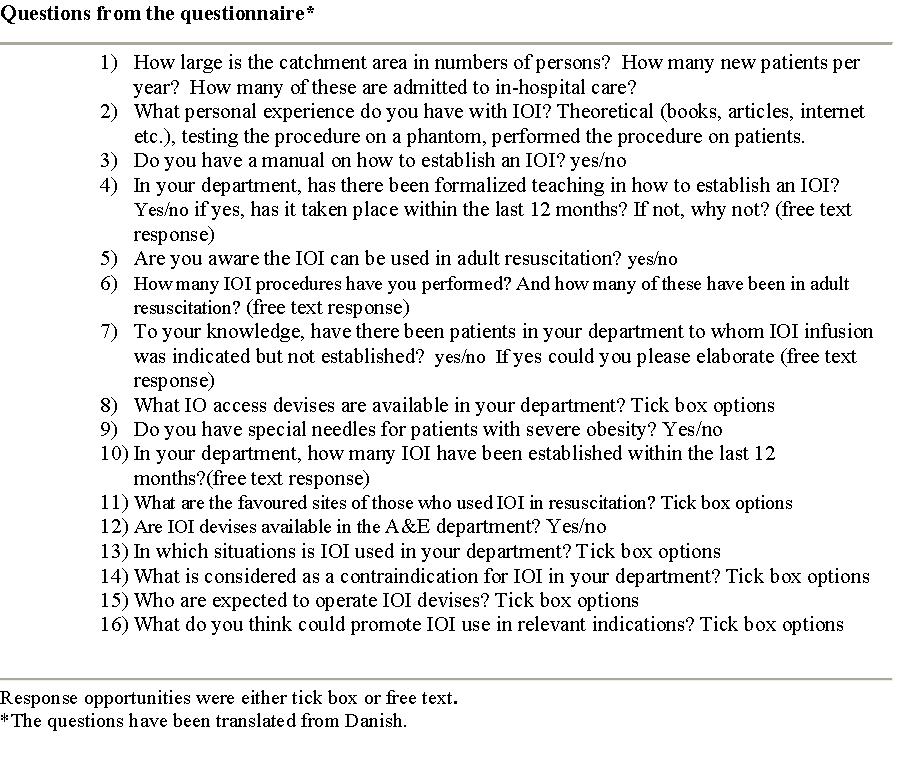

Supplement: Additional file 1 — Questions from the questionnaire. [file 1757-7241-18-37-S1.JPEG]
